# Two dimensional non-destructive testing data maps for reinforced concrete slabs with simulated damage

**DOI:** 10.1016/j.dib.2019.104127

**Published:** 2019-06-17

**Authors:** Harsh Rathod, Rishi Gupta

**Affiliations:** University of Victoria, Canada

**Keywords:** Non-destructive testing techniques, Ultrasonic pulse velocity, Infrared thermography, Ground penetrating radar, Half-cell potential, Electrical resistivity, Reinforced concrete structures

## Abstract

This research presents the use of a total of five Non-Destructive Testing Techniques (NDTs) and their combination to detect and quantify subsurface simulated defects in Reinforced Concrete slabs. The NDT techniques were applied on a total of nine 1800 mm × 460 mm reinforced concrete slabs with varying thicknesses of 100 mm, 150 mm and 200 mm. Contour data maps from each technique were prepared. This Data article presents the Non-Destructive Testing Techniques’ specifications, experimental set-up and converted 2-Dimensional NDT data maps for reinforced concrete slabs with simulated damage. The experimental research shows that combining multiple techniques together in evaluating the defects give significantly lower error and higher accuracy compared to that from a standalone test. For more details on the accuracy model of the NDTs, refer to the full length article entitled “Sub-surface simulated damage detection using Non-Destructive Testing Techniques in reinforced-concrete slabs” https://doi.org/10.1016/j.conbuildmat.2019.04.223 Rathod et al., 2019.

**Table:** Specifications table

Subject area	*Civil Engineering, Structural Engineering*
More specific subject area	*Non-Destructive Testing Techniques, Structural Health Monitoring*
Type of data	*Table and figures*
How data was acquired	*Several NDTs were used. The specifications of each instrument is included in*Table 1
Data format	*Raw and analyzed*
Experimental factors	*No-Pretreatment of test samples (All the NDTs were performed in the ambient environmental conditions. The slabs were subjected to the winter and summer cycles while being monitored. In Victoria, BC, Canada the temperature range during summer hours is 12–24 °C and during winter hours is 3–9 °C. These numbers are long-term historical averages based on climate data gathered from 1981 to 2010)*
Experimental features	*The data collected here includes more than 300 data points for each test slab. Total of 5 NDTs were used.*
Data source location	*Civil Engineering Materials Facility* *University of Victoria* *Victoria, BC V8N 5M8* *Canada* *Co-ordinates:* *48.469473, -123.309917*
Data accessibility	*The data is within this article.*
Related research article	*H. Rathod, R. Gupta, Sub-surface simulated damage detection using Non-Destructive Testing Techniques in reinforced-concrete slabs,* *Construction and Building Materials, Volume 215, 2019, Pages 754–764**[1]*

**Table:** 

**Value of the data** • The data maps presented here are of control (no defects) reinforced concrete slabs and slabs with sub-surface simulated damage. The maps highlight the comparison of different NDTs in detecting and quantifying damage. • The maps allow NDT practitioner in field to identify potential damage by correctly interpreting the NDT data. • This data maps will help researcher to develop similar experiments with different simulated damage to determine NDTs capability in detecting and quantifying sub-surface damage. • The work presented here is a foundation to interpret NDTs data correctly as it compares the individual data points of slabs with no defects and the slabs with simulated damage.

## 1 Data

Each data point collected from Reinforced concrete slabs with simulated damage and control slabs were converted into either intensity maps or contour maps to determine the performance of NDTs. Table 1 below shows the experimental setup and details related to data collection.

**Table 1:** NDTs specifications and experimental setup.

NDT technique	Equipment	Company	Specification	Experimental Setup
Ground Penetrating Radar (GPR)	Structure Scan Mini	GSSI	Max Depth = 50 cm Antenna Frequency = 2600 MHz	
Infrared Thermography (IRT)	E60	FLIR	IR Resolution = 320 × 240 pixels Spatial Resolution = 1.36 mrad Thermal Sensitivity = <0.05 °C	
Electrical Resistivity (ER)	Resipod	Proceq	Frequency = 40 Hz Resolution (nominal current 200μA) = ±0.2 kΩcm or ±1% (whichever is greater) Resolution (nominal current 50μA) = ±0.3 kΩcm or ±2% (whichever is greater) Resolution (nominal current < 50μA) = ±2 kΩcm or ±5% (whichever is greater)	
Ultrasonic Pulse Velocity (UPV)	Two Transducer Probes	Proceq	Resolution = 0.1 μs Bandwith = 54kHz	
Half Cell Potential (HCP)	Single Point Probe	Tinker and Rasor	Model – 6B Type = Copper–Copper Sulphate	

**Fig. 1:**
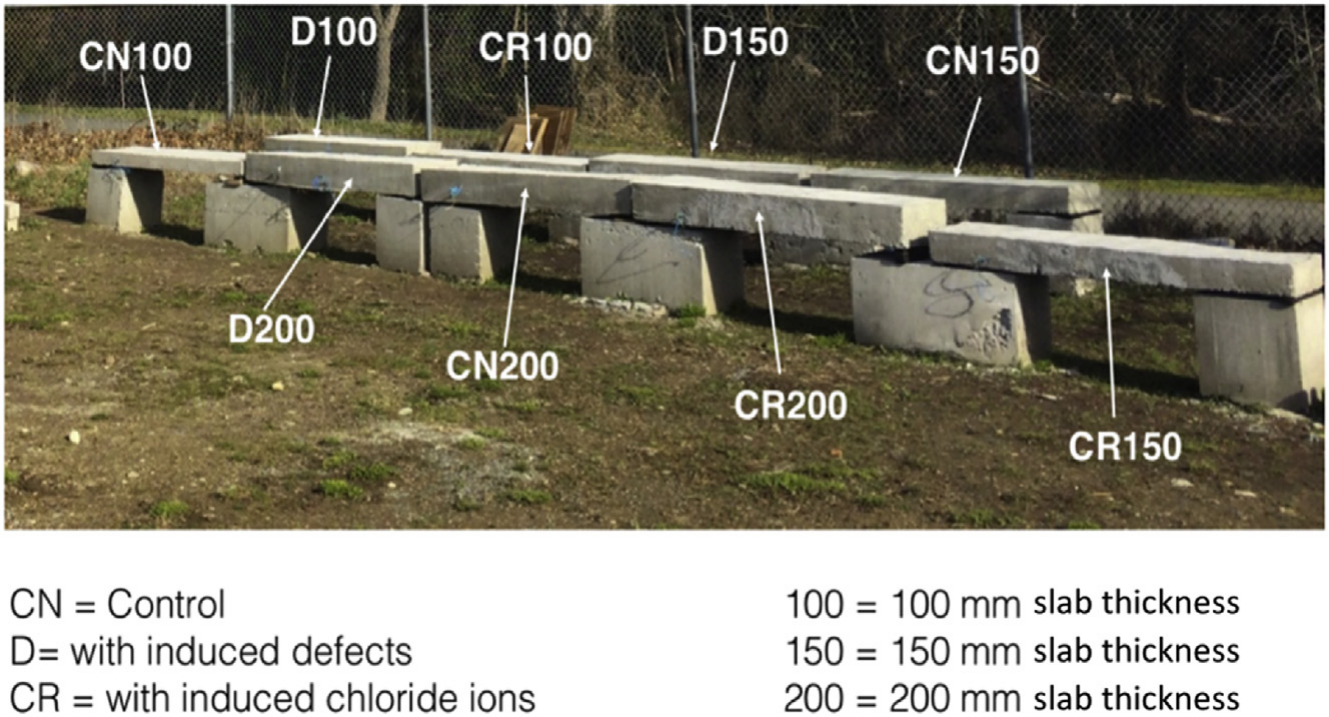
Experimental setup of the nine RC test specimens.

**Fig. 2:**
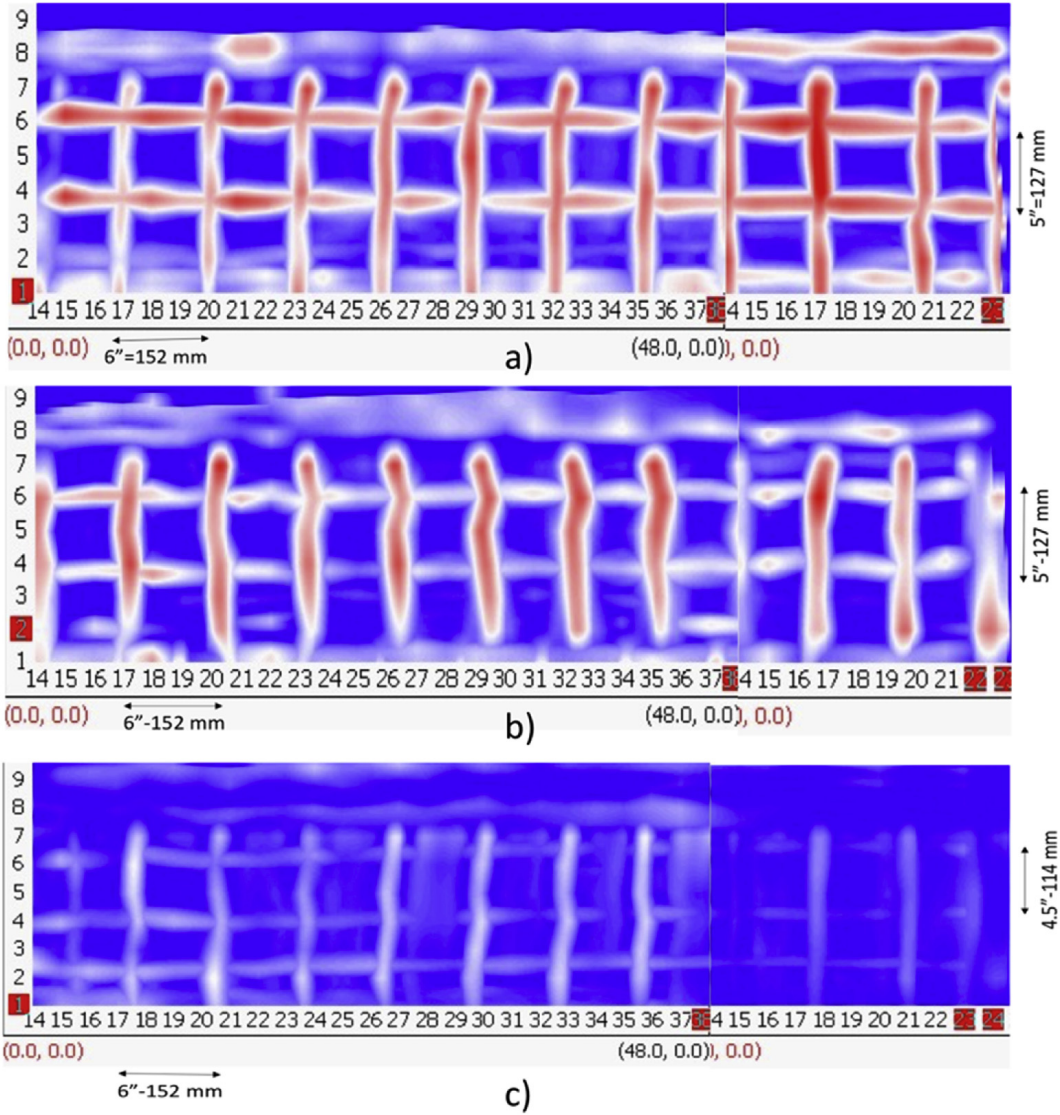
GPR Maps – Control Slabs - a) 100 mm, b)150 mm, and c) 200 mm.

**Fig. 3:**
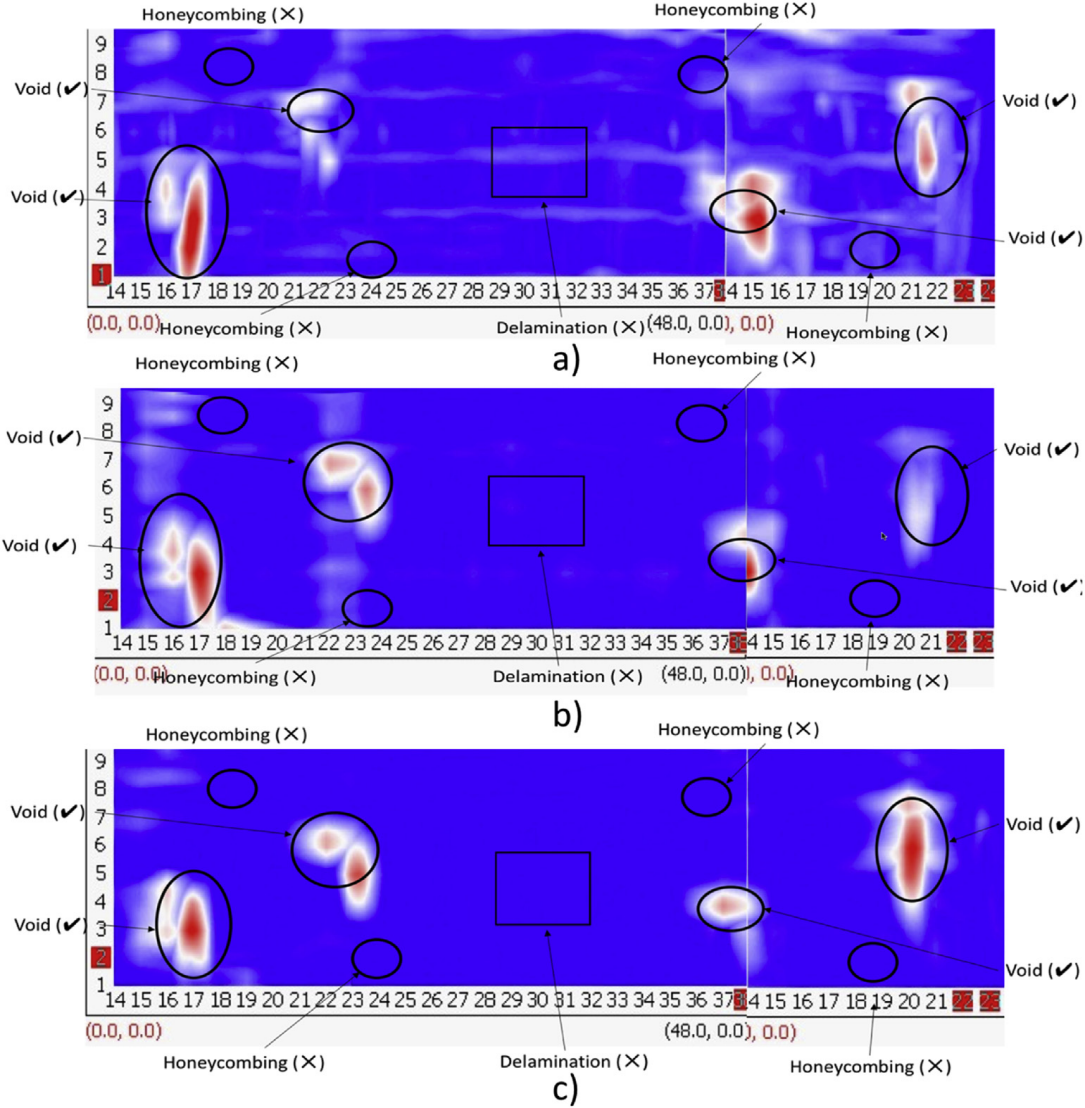
GPR Maps – Slabs with subsurface defects - a) 100 mm, b) 150 mm, and c) 200 mm.

**Fig. 4:**
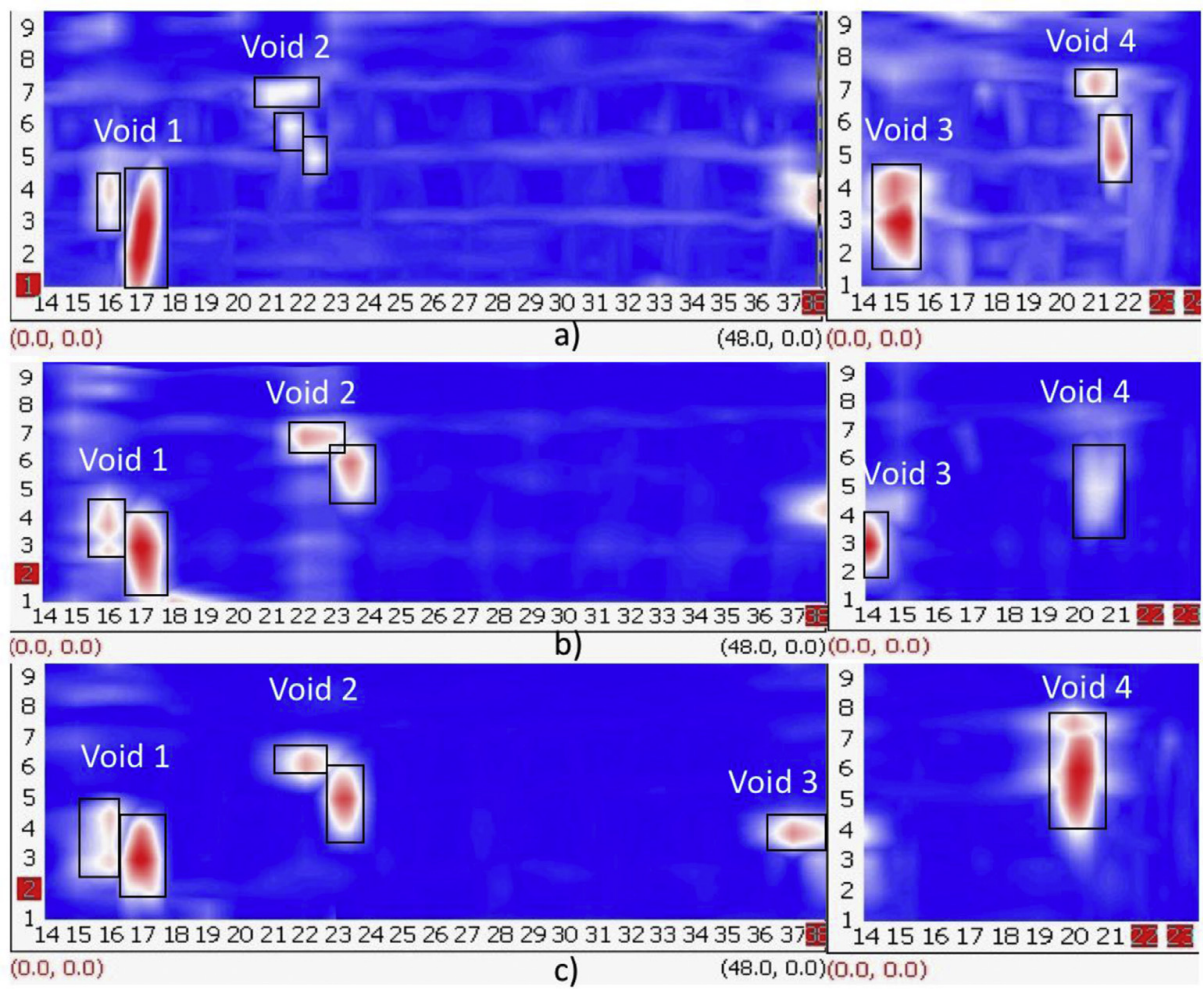
GPR- Area, Depth and Volume Accuracy Computation: a) 100 mm, b) 150 mm, and c) 200 mm.

The experimental set up is shown in Fig. 1, where all nine test specimens were placed together on 1.5 feet high concrete blocks to access the slabs from bottom as well if required in the future. Fig. 2, Fig. 3, Fig. 4 are data maps of Ground Penetrating Radar.

**Fig. 5:**
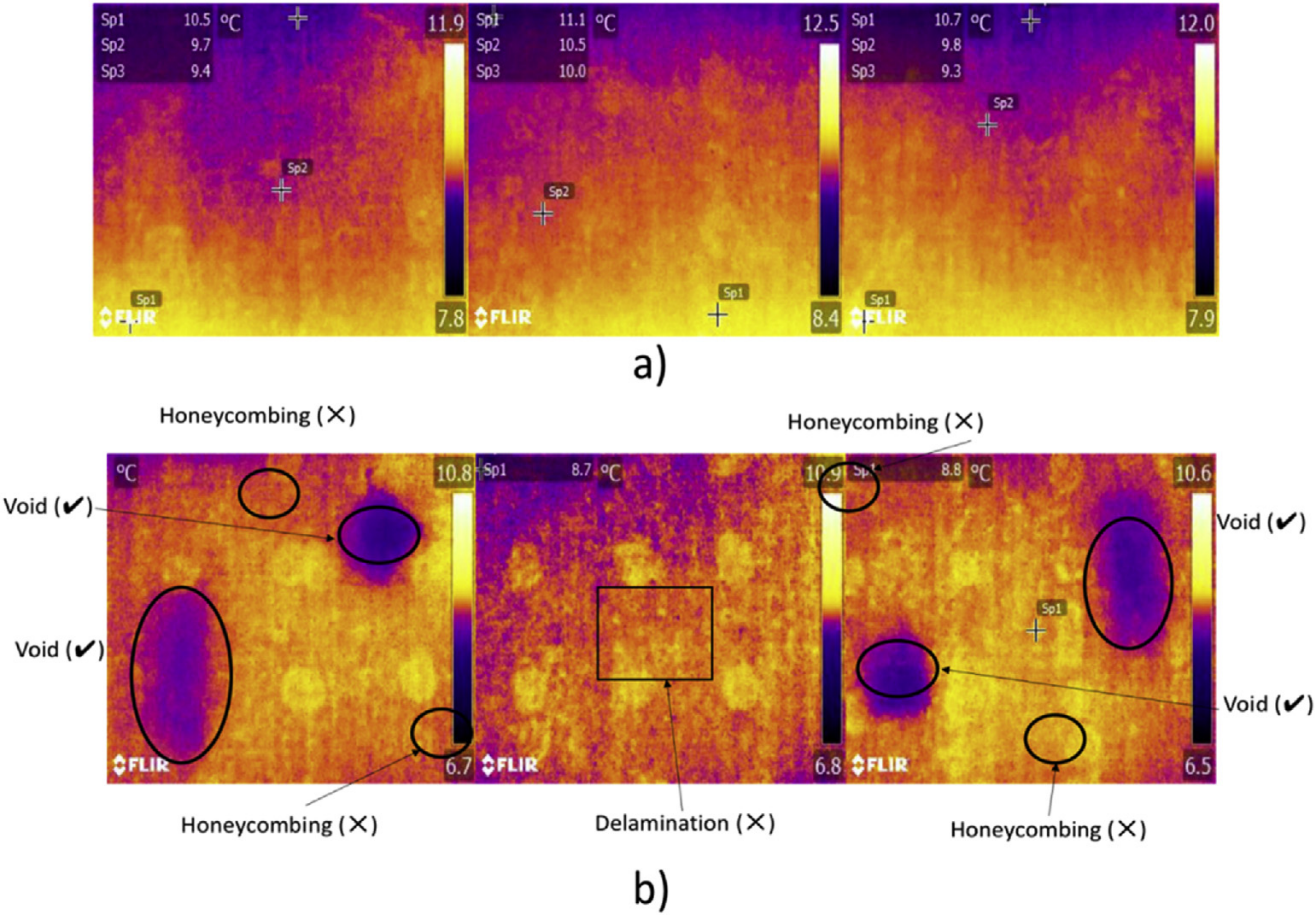
IR Thermographs – Control and Slab with Defects −100 mm.

**Fig. 6:**
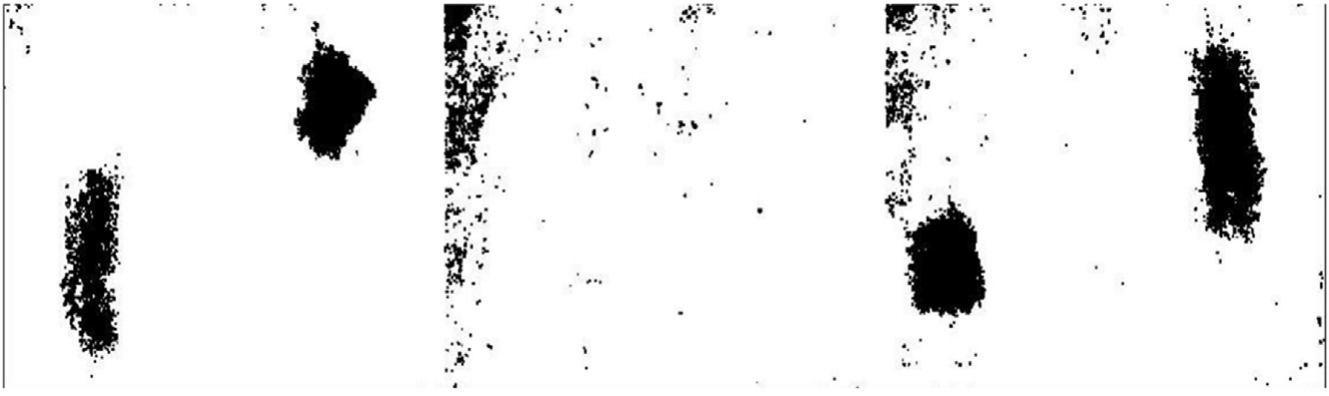
IRT- Area Accuracy Computation:100 mm slab using MATLAB Software.

Fig. 5 is an Infrared Thermograph captured to identify temperature difference between the embedded voids and surrounding sound concrete. Fig. 6 shows the processed map to compute the area of voids.

**Fig. 7:**
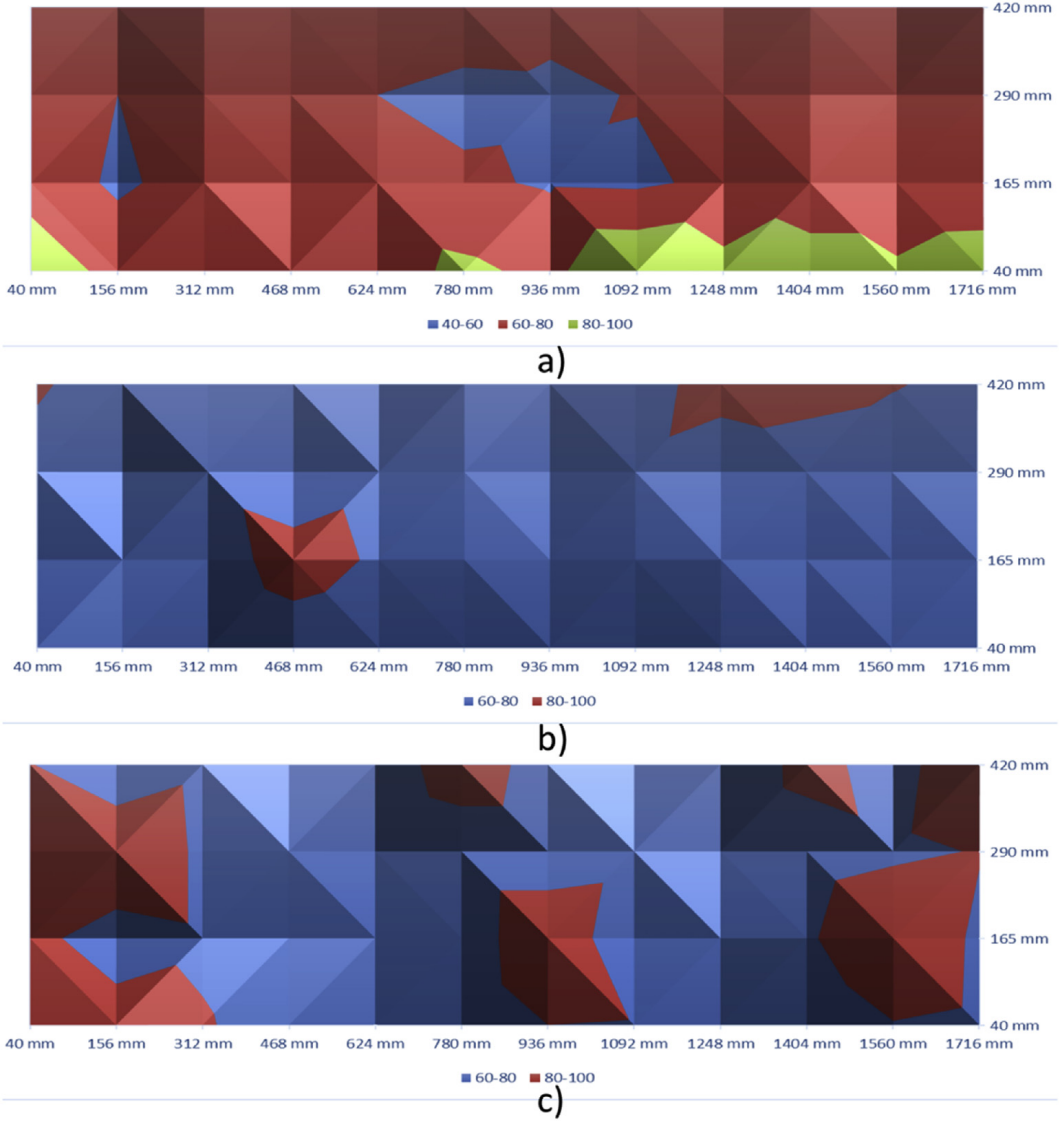
ER Contour Maps - Control Slabs - a) 100 mm, b)150 mm, and c) 200 mm.

**Fig. 8:**
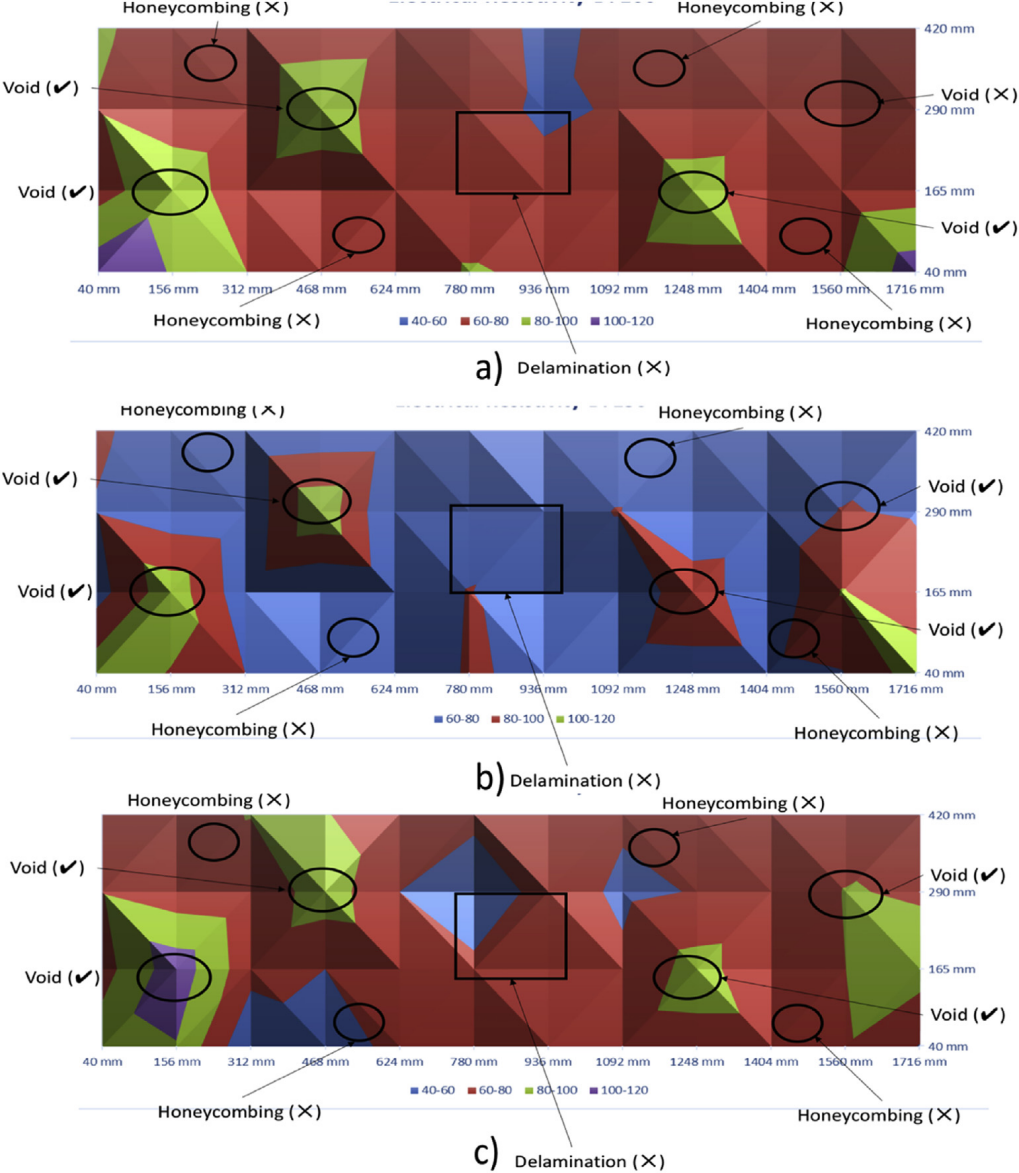
ER Contour Maps- Slabs with subsurface defects - a) 100 mm, b)150 mm, and c) 200 mm.

Fig. 7, Fig. 8 are Electrical resistivity contour maps produced in Microsoft Excel.

**Fig. 9:**
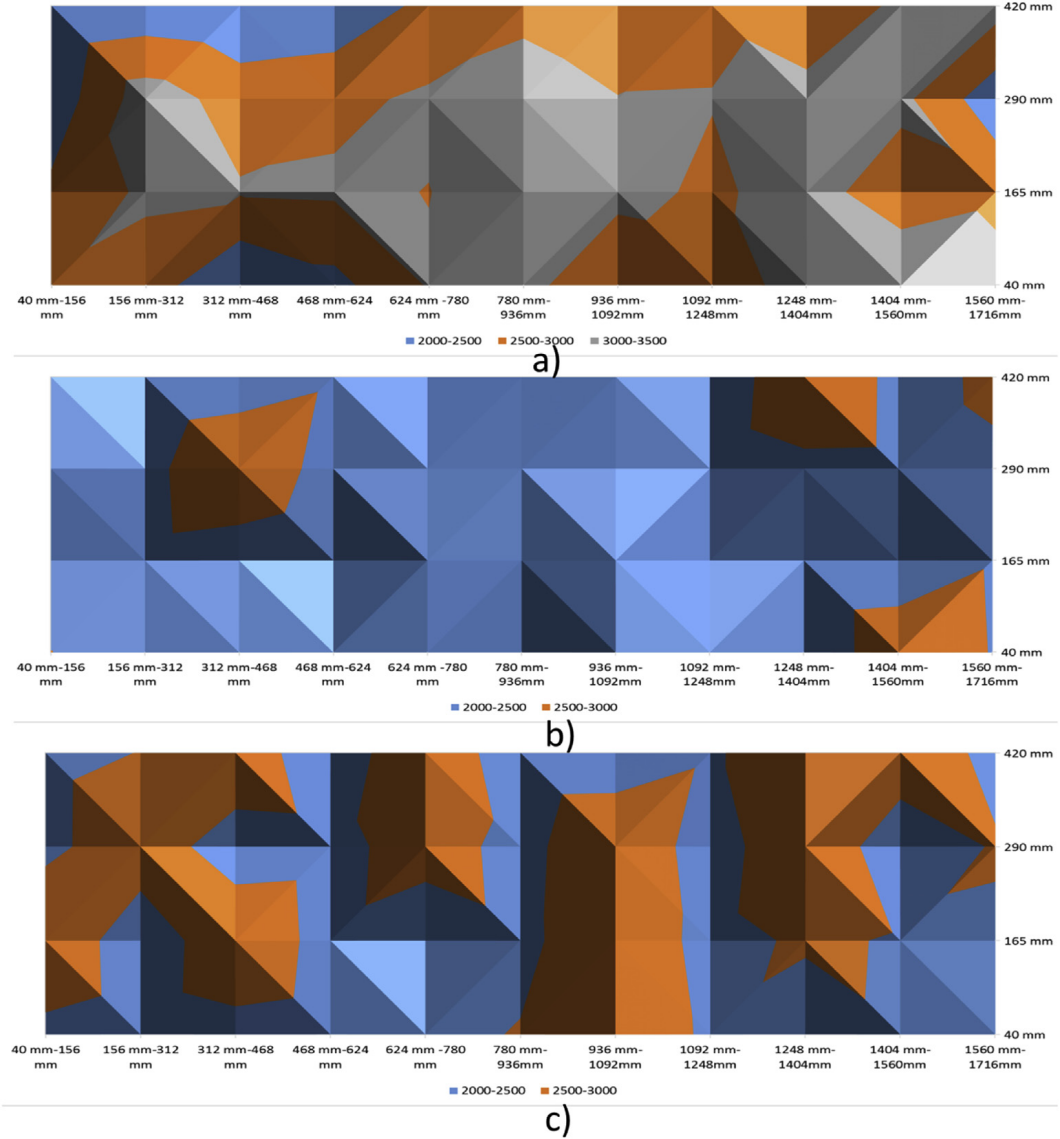
Ultrasonic Pulse Velocity-Control Slabs - a) 100 mm, b)150 mm, and c) 200 mm.

**Fig. 10:**
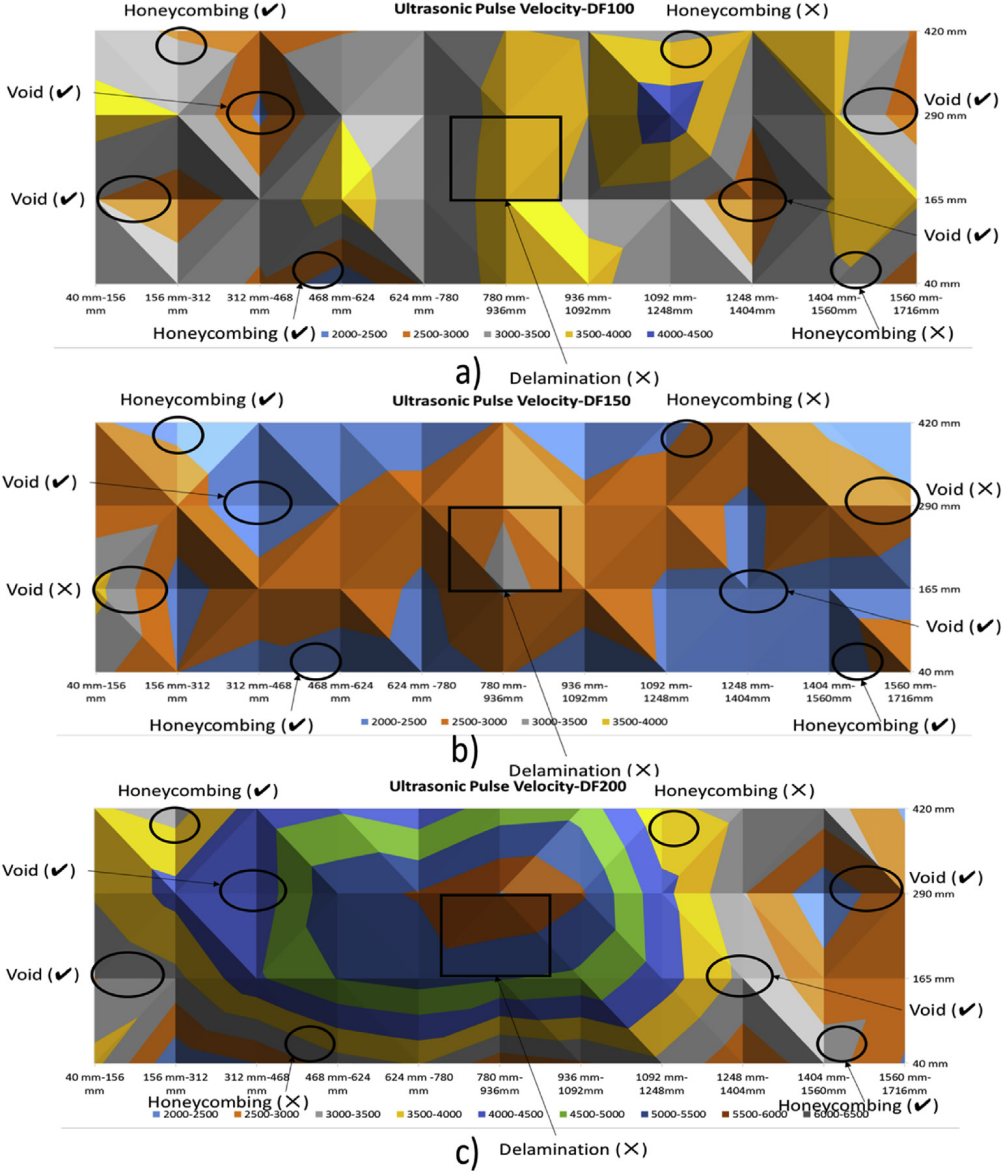
Ultrasonic Pulse Velocity-slabs with Defects - a) 100 mm, b)150 mm, and c) 200 mm.

Fig. 9, Fig. 10 show the contour maps produced using the data obtained from Ultrasonic Pulse Velocity. These maps are for 100 mm, 150 mm and 200 mm slabs (both control and with defects).

**Fig. 11:**
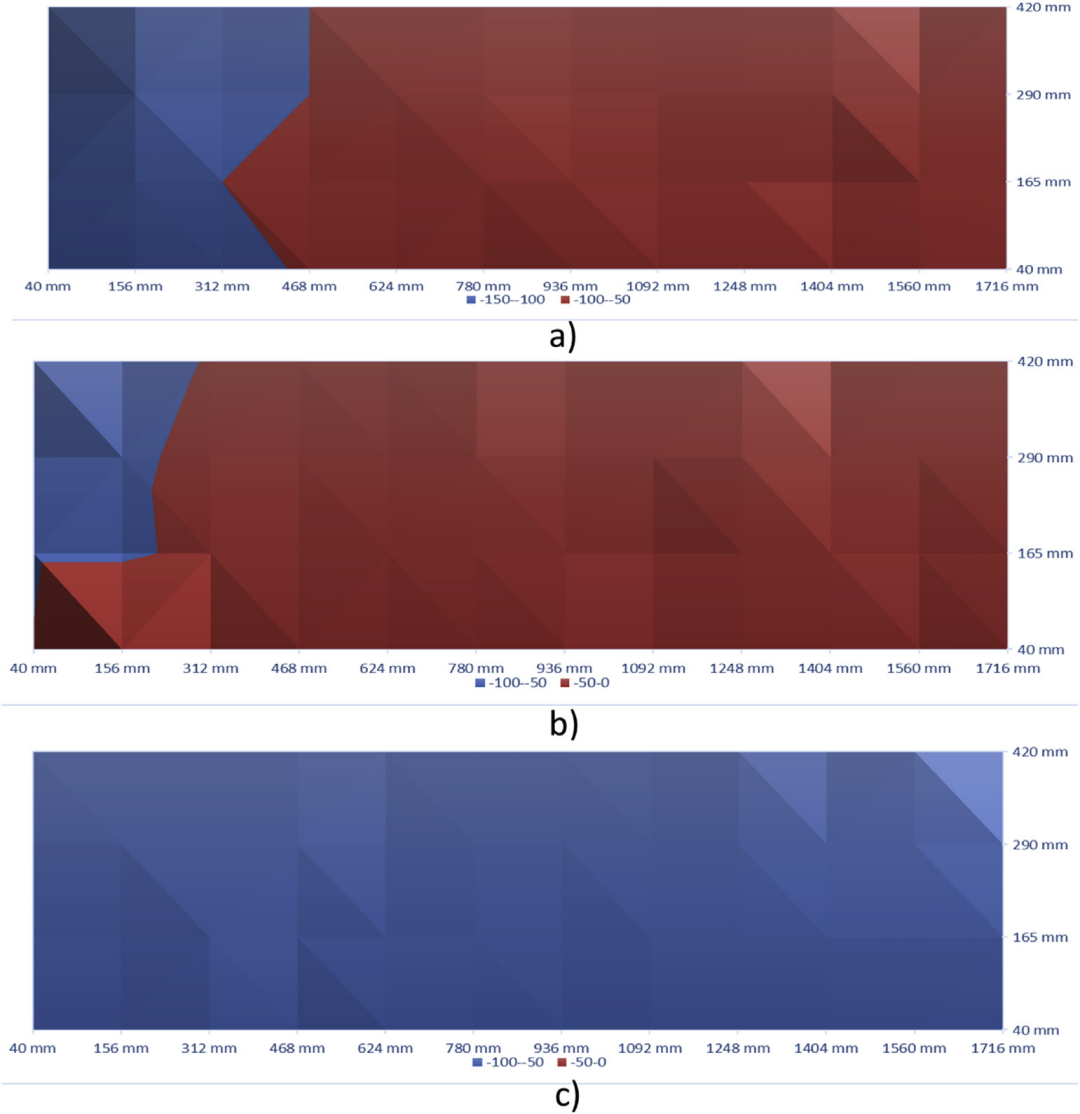
Half-Cell Potential - Control Slabs - a) 100 mm, b)150 mm, and c) 200 mm.

**Fig. 12:**
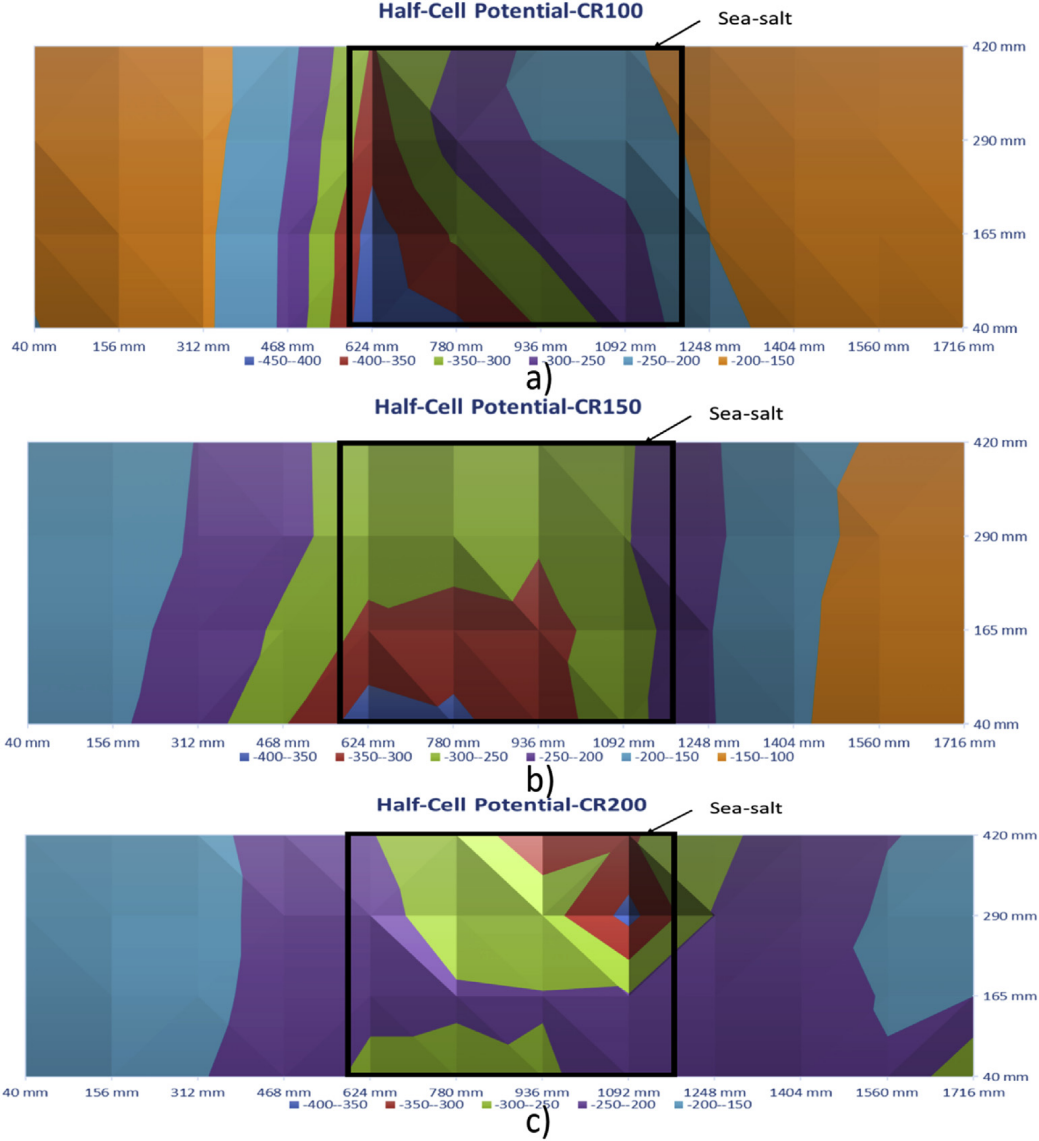
Half-Cell Potential-Corrosion Slabs - a) 100 mm, b)150 mm, and c) 200 mm.

Fig. 11, Fig. 12 show the contour maps produced using the data obtained from Half-Cell Potential Technique. These maps are for 100 mm, 150 mm and 200 mm slabs (both control and with defects).

## 2 Experimental design, materials and methods

As shown in Table 1, a hand held FLIR E60 camera was used to acquire infrared thermographs of the test specimens. The acquisition distance was kept as 3 ft. (about 0.9 m) constant to the top surface of the slabs so as to cover the third portion of the slab. Total three thermographs per each slab were captured to cover the entire slab. Thermographs were taken only of the top surface of the slabs.

For the UPV test, two transducers and a Data Acquisition (DAQ) System from Proceq were used to collect indirect data from the test specimens. As highlighted in the introduction section, indirect transmission is not an accurate method of measurement however, it is the most feasible. The transducers having a frequency of 54 kHz were used in this study. Both the transducers were kept approximately 130 mm apart on the rebar grid points (longitudinal and transverse rebar junction points) to obtain the velocity values of the RC slabs. This resulted in a total of 44 points per slab.

For measuring the surface electrical resistivity of the RC test slabs, four-point Wenner probe setup (Resipod) from Proceq was used.

In order to measure the corrosion potential of the RC slab, a copper-copper sulphate probe called half-cell was used along with a voltmeter. Measurements were taken on the same grid of 132 mm × 156 mm as used for the UPV and ER. It should be noted that the chosen density of readings is quite high. This is in order to enable establishment of a good correlation between the techniques.

GPR equipment- StructuresScan Mini from GSSI (Geophysical Survey Systems, Inc.) requires finer grid/mesh when scanning the RC elements. A mesh size of 2 inches × 2 inches (50 mm × 50 mm) was used when collecting the data which resulted in a total of 15 scans for both the directions.
